# Prevalence of onchocerciasis in the Fundong Health District, Cameroon after 6 years of continuous community-directed treatment with ivermectin

**Published:** 2011-11-10

**Authors:** Henri Lucien Fouamno Kamga, Dickson Nsagha Shey, Jules Clement Nguedia Assob, Anna Longdoh Njunda, Peter Nde Fon, Peter Kindong Njem

**Affiliations:** 1 Department of Medical Laboratory Sciences, Faculty of Health Sciences, University of Buea, Cameroon; 2 Department of Public Health and Hygiene, Faculty of Health Sciences, University of Buea, Cameroon; 3 Department of Nursing, Faculty of Health Sciences, University of Buea, Cameroon; 4 Medicine programme, Faculty of Health Sciences, University of Buea, Cameroon

**Keywords:** Onchocerciasis, microfilaria, district, ivermectin, Cameroon

## Abstract

**Introduction:**

Onchocerciasis is one of the leading infectious causes of blindness affecting over 37 million people of which 99% are in Africa. The purpose of this study was to determine the prevalence of onchocerciasis in the Fundong Health District, a locality where community-directed treatment with ivermectin has been carried out for 6 consecutive years.

**Methods:**

Questionnaires covering participants’ identity, Rapid Epidemiological Assessment (REA) for onchocerciasis and parasitological parameters were distributed to participants. Skin snip (SS) was collected for laboratory investigation.

**Results:**

A total of 404 participants belonging to 200 households were randomly selected from the Fundong Health District, of which 134 (33.2%) were males and 270(66.8%) were females, 14 (3.5%) had microfilaredermia and 15(3.7%) had nodules. There was no significant difference in the prevalence of microfilaredermia with respect to age of participants (X^2^=2.749, P=0.601). There was however a statistically significant difference in the prevalence of nodule and impaired vision/eye itching (IVIE) with respect to age (X^2^=24.67, P<0.001). The greatest rate of infection was found among farmers (2.5%) followed by students (0.7%) and businessmen (0.25%).

**Conclusion:**

This study shows that the study area is now hypo-endemic for onchocerciasis, following 6 years of continuous treatment with ivermectin. Careful monitoring of onchocerciasis should however be continued to avoid that the area returns to its initial hyper endemicity.

## Introduction

Onchocerciasis, commonly known as river blindness is a parasitic infection caused by the lymph space and subcutaneous-dwelling *Onchocerca volvulus*. The disease is transmitted by black flies of genius *Simulium*. It produces untold hardship characterized by severe pruritus, formation of nodules (onchocercomas), changes in skin pigmentation, loss of skin elasticity, revolting fleshy deformities (hanging groin or adenolymphocele), impaired vision and blindness due to invasion of the eyes by microfilaria. The disease remains a major public health and socioeconomic problem to the African continent [1].

Onchocerciasis is one of the leading infectious causes of blindness in Africa [2–4]. For many years, onchocerciasis was thought to have affected 17 million people [5]. Recently, this figure was estimated to have risen to more than 37 million [6] as more attention was paid to remote areas in Central African countries. According to WHO [7], onchocerciasis has not caused a single death but the global burden is 987,000 disability adjusted life years (DALYs). The disease has been found to significantly reduce the lifespan of infected people. Nigeria, Chad, Sudan, Central African Republic and Cameroon register high incidences where more than 60% of the population is infected [8].

In 1995, the WHO launched the African programme for Onchocerciasis Control (APOC) that was intended to eliminate onchocerciasis from the African continent through Community Directed Treatment with Ivermectin (CDTI) in the next 12years [9]. However following the 2006 and 2007 progress report, the APOC was extended to 2015 [10].

Rapid Epidemiological Mapping for Onchocerciasis, (REMO) has shown that the disease is endemic in Cameroon [11,12]. Rapid Epidemiological Assessment(REA) for onchocerciasis and Rapid Assessment Procedures for Loiasis (RAPLOA) [13,14] have been used in combination to ensure safe and effective CDTI, owing to serious adverse events (SAEs) noticed in *Loa loa* and *Onchocerca volvulus* co-endemic areas [15–17]. The only current control measure is CDTI [18]. In this perspective the purpose of this study was to determine the prevalence of onchocerciasis in a region where CDTI has been carried out for at least 6 consecutive years, in order to evaluate the impact of ivermectin treatment in an initially hyperendemic area, where more than 45% of the population was infected [19], and extrapolate to other locations with similar treatment profiles.

## Methods

### Study area

Fundong Health District (FHD) is one of the 18 Health Districts in the North West Region (NWR) of Cameroon. It covers a surface area of 145km^2^. FHD occupies about 4/5th of the land surface area and population of Boyo Division. It is located between latitude 6° 4'and 6°23’ to the North of the equator and longitude 10° and 10° 33’ to the East of the Greenwich Meridian. The District headquarter, Fundong, is about 69 km from Bamenda, the Regional headquarter, linked by a tarred road. The District is generally hilly with most of the towns located on hills and valleys. It has typical savannah grassland vegetation and two distinct climates; the rainy season and dry season. The rainy season which begins from late March to early November is characterized by thunder storms, lightening, hailstones and torrential rains. The dry season that extends from mid-November to mid March and is characterized by cold chilly winds.

### Primary visits and participant recruitment

The local authorities and the community leaders were earlier visited and presented with the project as their involvement was a key factor for its success. All the houses in the Health District were allocated a number. 200 houses were selected at random, by balloting. This sampling approach has already been used elsewhere [20].

The randomly selected households were visited and presented the project. They were however explained that it was not an obligation for them to participate in the research and neither was it a pre-requisite to accessing routine medical or other social services publicly available. Ethical clearance was obtained from the Regional Delegation of Public Health. Written informed consent forms were distributed and explained, and only members aged between 15 and 85 years in selected households, who voluntarily accepted to sign and return the consent form were involved in the study.

### Data collection

Information was obtained from participants by the use of questionnaires. The questionnaire was designed in three parts covering participants’ identity, Rapid Epidemiological Assessment (REA) for onchocerciasis and parasitological parameters. Skin snip (SS) specimen was collected from every participant. No special collection time was observed as the microfilariae of *Onchocerca* are non-periodic. Procedures for collection and processing of skin snip (SS) are described elsewhere [8]. Each sample collected was immersed in normal saline solution in the well of a microtitration tray and the tray was placed on a piece of damp tissue in a Petri dish to prevent the preparation from drying out. Samples collected in a household were sent to the hospital laboratory before we visited the next household.

### Statistical analysis

The statistical packages, Epi-Info 6.04 (CDC, 2001) and SPSS version 17.0 were used to analyze the results obtained. Chi-square (X^2^) test differences were considered significant at P<0.05.

## Results

A total of 404 participants belonging to 100 households were randomly selected from a map of the village. The age range of the study population was 15 to 85 years (median, 41 years); 66.6% were females and farmers represented 44.1%. Majority (87.4%) of the participants had a history of ivermectin treatment. The overall prevalence of Microfilaredermia was 3.5% (2.0% in males and 1.5% in females). Three hundred and forty one (84.4%) participants reported to have had some troublesome itching of their bodies either currently or in the past of which 67.5% were either visually impaired and/or have had a history of itching of the eyes.

Table 1 shows the prevalence of microfilaredermia, nodules and IVIE in different age groups. There was no significant difference in prevalence of microfilaredermia with respect to age (X^2^=2.74, P=0.601). The highest prevalence of nodules (2.7%) and IVIE (11.9%) was among participants aged above 56 years and there was a statistically significant difference in the distribution of nodules (X^2^=24.67, P^2^= 19.87, P=0.003) among the different age groups.

**Table 1:** Prevalence of microfilaredermia, nodules and impaired vision/eye itching (IVIE) in different age groups in the Fundong Health District, Cameroon

Age groups	Number (%) of subjects with microfilaria(e)	Number (%) of subjects with nodules	Number (%) of subjects with IVIE
15-25 (n=91)	3 (0.7)	0 (0.0)	43 (10.6)
26-35 (n=80)	2(0.5)	1(0.2)	33 (8.2)
36-45 (n=77)	1(0.2)	2(0.5)	36 (9.0)
46–55 (n=67)	3(0.7)	1(0.2)	39 (10.0)
56 and above (n=89)	5 (1.2)	11 (2.7)	48 (11.9)
Total (n=404)	14 (3.5)	15(3.7)	199 (49.3)

Table 2 shows the prevalence of microfilaredermia, nodules and IVIE according to participants’ occupation. There was no significant difference in the prevalence of microfilaredermia with respect to occupation (X^2^=6.22, P=0.10). The highest prevalence of nodules (3.0%) and IVIE (24.8%) was among farmers and there was a significant difference in the distribution of nodules (X^2^=8.77, P=0.03) and that of IVIE (X^2^= 16.00, P=0.014) among the different occupational groups.

**Table 2:** Prevalence of microfilaredermia, nodules and impaired vision/eye itching (IVIE) according to participants’ occupation in the Fundong Health District, Cameroon

Occupation	Number (%) of subjects with microfilaria(e)	Number (%) of subjects with nodules	Number (%) of subjects with IVIE
Farmers (n=178)	10 (2.5)	12 (3.0)	100 (24.8)
Student (n=75)	3 (0.7)	0(0.0)	38 (9.4)
Business (n=87)	1 (0.2)	2 (0.5)	35 (8.7)
Civil servants (n=64)	0 (.0)	1 (0.2)	28 (7.0)
Total (n=404)	14 (3.5)	15 (3.5)	199 (49.3)

## Discussion

This study describes the current situation of onchocerciasis in the population of Fundong Health District in Cameroon after 6 years of continuous CDTI. The baseline data on the hyper -endemicity of the area 7 years back was obtained from the 2006 annual health report of the Fundong Health District [19]. Participants in this study were selected to be above 15 years of age to maximize the likelihood of involving only people who have been in the CDTI programme for the past 6 years, as the purpose of the study was to evaluate the impact of ivermectin treatment in the area.

This study shows a prevalence of 3.5% for onchocercal filariasis and demonstrates a transition from hyper to hypo-endemicity following 6 years of continuous treatment with ivermectin. The level of endemicity has fallen below 7%, the threshold presented to the APOC by Erasmus University researchers [10]. The low prevalence of onchocercal microfilaredermia in the study area despite the presence of abundant fast flowing rivers, black flies and an atmosphere that favours the interaction between farmers and biting flies can more likely be attributed to continuous CDTI. Consultation of the FHD CDTI registers suggests a mean annual therapeutic coverage of 72.5% form 2004 to 2010 (62.4% in 2004, 59.0% in 2005, 63.1% in 2006, 72.3% in 2007, 80.4% in 2008, 85.2% in 2009 and 2010 respectively). One may argue to attribute the low prevalence of the disease to the wide use of doxycycline by the community members as this drug is locally available and easily obtained from street vendors at low cost, and regularly prescribed for the treatment of chlamydiasis and other sexually transmitted infections which are among the first five causes of morbidity in this area [21]. Even though doxycycline has been shown to treat onchocerciasis by killing *Wolbachia* endobacteria of *Onchocerca volvulus* [22,23], about 4–6 weeks intensive treatment is needed to kill adult worms [24]. Therefore, despite the fact that some participants had a recent history of sexually transmitted infections and doxycycline treatment, the wide and indiscriminate use of doxycycline in the area could not have accounted for the drop of prevalence of onchocerciasis.

Prevalence of onchocercomas of 3.7% was observed. These cutaneous nodules were concentrated mainly around and below the waist which is typical of African form of onchocerciasis [25]. All individuals with nodules had a history of ivermectin treatment. This prevalence of onchocercomas might have been underestimated since not all nodules are usually palpable as some adult worms lie in single masses in the tissues [26]. Majority of people who had microfilaredermia, nodules and IVIE were aged above 55 years. This suggests that nodules formation is a characteristic of chronic infections and can be linked to intensity and level of exposure [26]. This also holds true in farmers as they had the highest prevalence of onchocercomas and IVIE.

Despite the low prevalence of microfilaredermia a vast majority (84.4%) of study participants complained of frequent skin itching. Host reactions to dead microfilaria that usually account for most of the pathogenesis [27] and side effects of ivermectin treatment, including pruritus [28], may not be enough account for the observed itching in the study population. Therefore, there can be other underlying causes of itching apart from onchocercal infection, such as allergic reactions in the dermis of the skin, fungi or bacterial infections.

Limitations of this study reside the failure to measure the prevalence of infection among ivermectin treated and non-treated individuals and the selection of participants; some of those who did not normally respond to previous treatment might not have accepted to sign the consent form.

## Conclusion

This study shows that the study area is now hypo-endemic for onchocerciasis, following 6 years of continuous treatment with ivermectin. Health information, sensitization, mobilization and advocacy will be quite instrumental in creating awareness on the various ways of transmission and how possible the disease can bounce back if neglected. Constant epidemiological surveys are needed to monitor the disease in its hypo-endemic state. Further findings are needed to determine the prevalence of other filarial species and in this area.
